# Bulk storage of mango (Mangifera indica L.) and pineapple (Ananas comosus L.) pulp: effect of pulping and storage temperature on phytochemicals and antioxidant activity

**DOI:** 10.1002/jsfa.9762

**Published:** 2019-05-30

**Authors:** Palitha C Arampath, Matthijs Dekker

**Affiliations:** ^1^ Food Quality and Design Group, Department of Agrotechnology and Food Sciences Wageningen University Wageningen The Netherlands; ^2^ Department of Food Science & Technology, Faculty of Agriculture University of Peradeniya Peradeniya Sri Lanka

**Keywords:** antioxidant, bulk storage, health promoting, kinetics, mango, pineapple

## Abstract

**BACKGROUND:**

The effects of pulp extraction, thermal treatment and bulk storage of mango (Mangifera indica L.) and pineapple (Ananas comosus L.) pulps for 20 weeks at ambient (28 ± 2 °C) and cold (4 °C) temperatures on the bioactive phytochemicals and antioxidant activity were investigated.

**RESULTS:**

The contents of total polyphenols in mango (10.5%) and pineapple (5.4%) increased during pulping. The ratio of the degradation rate constants (*k*_d_ values) (28 ± 2 °C: 4 °C) of vitamin C, polyphenols, Trolox equivalent antioxidant capacity (TEAC) and β‐carotene ranged from 2–4.5 and 1.5–2.7 in mango and pineapple pulps, respectively. The *k*_d_ values of tannic acid, chlorogenic acid, epicatechin and catechin in mango pulp were 1.5–1.8 times higher under ambient storage than in cold storage. Furthermore, in pineapple pulp, the degradation rates of the same components were 1.6, 1.6, 2.1 and 1.4 times, respectively, faster at room temperature than in cold storage. The bulk storage of pulps at 4 °C provided better retention of health‐promoting compounds than ambient temperature storage for up to 20 weeks.

**CONCLUSION:**

Bulk storage of mango and pineapple pulp under cold storage conditions (4 °C) is recommended as a better pulp preservation method than storage at ambient (28 ± 2 °C) temperature. © 2019 The Authors. *Journal of The Science of Food and Agriculture* published by John Wiley & Sons Ltd on behalf of Society of Chemical Industry.

## INTRODUCTION

Mango (*Mangifera indica* L.) and pineapple (*Ananas comosus* L.) are commercially important tropical fruits in the world fruit trade. Mature or ripe fruits are consumed fresh or in processed forms, including as juice, nectar, canned and dehydrated products. Mango is a tropical fruit that is cultivated in many countries with a tropical climate, including Sri Lanka, hence its global importance. Mango is rich in antioxidants such as carotenoids, ascorbic acid and various polyphenolic compounds.^1^ In Sri Lanka, the peak season for mango is from mid‐April to late‐June, and a second low production peak is evident from mid‐November to mid‐January. There is a high demand in the local and foreign markets for fresh fruits and value‐added products. Bulk storage of the pulp or juice is a common practice of the fruit processors in Sri Lanka to preserve raw material for continuous production during offseason.

Pineapple belongs to the family Bromeliaceae, which consists of approximately 2000 species. It is the eighth most abundantly grown fruit in the world, with over 14 million tons produced annually.^2^ Fresh ripe pineapple fruits and processed products are rich in aroma and flavour compounds. The phenolic profile of pineapple juice and the broader physicochemical characterization of pineapple have been reported by Luximon‐Ramma *et al*.^3^. and Brat *et al*.^4^. In Sri Lanka, the peak season of pineapple is from April to June, and a second low production peak occurs from December to mid‐January. The development and application of a low‐cost preservation technique for fresh fruits that retains the nutritional constituents would be an important way to reduce fruit waste.^5^ Mango and pineapple pulp are commercially produced and stored during the peak harvest seasons. This stored pulp is then used as a raw material in the beverage, bakery, dairy and confectionery industries. Therefore, pulp is a versatile semi‐processed product that can be converted to different value‐added products. Although there is a high demand for frozen pulps without preservatives in the international markets, freezing is not cost effective in some countries, such as Sri Lanka.

Industrial preparation of pulp consists of several stages of heat treatment for steam‐peeling or blanching, pulping, thermal inactivation of endogenous enzymes, and pasteurization of the pulp or puree followed by hot filling and packaging. Inactivation of the oxidative enzymes after disintegration of the fruit flesh is a vital prerequisite to prevent browning of the pulp, puree and beverages. For preservation at ambient temperatures, mainly weak organic acids, such as benzoic, acetic, sorbic, and propionic acids, and sulphites are commonly added alone or in combinations at concentrations up to 0.1% (by food weight).^6^

In fruit and vegetable tissues, the naturally available carotenoids are stabilized by the plant matrix. Mechanical processes (cutting and pulping) disrupt the tissues and expose them to oxygen and endogenous oxidative enzymes, provoking oxidation reactions.^7^ Bulk storage of fruit pulp is a common practice in the fruit processing industries in Sri Lanka. Thermal treatment, followed by the addition of preservatives and storage at ambient (28 ± 2 °C) or cold (4 °C) temperatures, are common operations in Sri Lanka. Industrially preserved pulp can be stored for 6 months at ambient temperature or 10 months under cold storage (4 °C). These practices are relatively low‐cost and affordable to small‐ and medium‐scale fruit processors as a strategy for preserving fruit pulp. The availability of fruits during the season is limited to a few weeks to up to 1 to 2 months. However, consumer demand for the products derived from these fresh fruits or preserved pulp persists throughout the year.

In a typical industrial process, the treatment condition of fruit pulp is basically 85 ± 1 °C for 20 to 25 min where the jacket temperature of the steam jacketed container was maintained at 100 °C. Thermally treated pulp was cooled to 30 °C and treated with potassium metabisulphite (KMS).

This treatment is sufficient to inactivate polyphenol oxidase and destroy spoilage microorganisms.^8^ The treated pulp is transferred into pre‐cleaned, sterilized plastic drums, sealed air tightly and stored for later use to convert to value‐added products. Although the pulp is preserved with these practices, the effects of processing and storage temperatures on the health‐promoting phytochemicals and antioxidant activity during the bulk storage of mango and pineapple pulp have not been investigated. Such information would be useful for industrialists, process designers, quality controllers, nutritionists and consumers.

Therefore, the objective of this research is to determine the effect of the pulping and storage temperatures on the phytochemicals and antioxidant activity of mango and pineapple pulps and to determine the degradation kinetics of the health‐promoting compounds during bulk storage at cold (4 °C) and ambient (28 ± 2 °C) storage temperatures.

## MATERIALS AND METHODS

### Mango and pineapple pulp production and storage

Industrial‐scale processing experiments were conducted at a leading fruit processing factory in Sri Lanka. The maturity of the fruit lot was judged based on the peel colour and flesh colour around the seeds in randomly selected fruits using the standard colour charts for maturity determination available in the processing plant. The fully mature stage is indicated by the peel colour changing from dark green to light greenish yellow. Yellow‐coloured flesh around the seeds is an indicator of the fully mature stage, while white flesh is an indicator of the immature stage. Selected mature mangoes were stored for 6 days for ripening. The peel colour of pineapple fruits gradually changes from dark green to light green yellow or sometimes to a deep orange at the onset of maturity. Fruits harvested at colour break and 20% yellow stage can be kept for 10 days at ambient temperature (28 ± 2 °C).

Mangoes collected from the suppliers were manually sorted to remove damaged, immature, over‐ripe, cut and bruised fruits. The selected fruits were washed, peeled and cut into pieces before feeding into a pulping machine (Robot Coupe‐C120‐1HP Commercial Juicer/Pulp Extractor, Ridgeland, MS, USA). Pulp separated from the machine was thermally treated at 85 ± 1 °C for 20 to 25 min in an industrial‐scale steam‐jacketed kettle (Lee Inc. 125‐Style, 125‐gal capacity, Philipsburg, PA, USA) at 100 °C with an agitator. Sodium metabisulphite (SMS) (50–60 g SMS/60 kg pulp weight) was added after the thermal treatment and mixed with the pulp. The heat‐treated mango and pineapple pulps with added preservatives were hot filled into high‐density polyethylene (HDPE) bags in plastic containers (50 kg capacity). The bags were hermetically sealed, labelled and stored in cold storage at 4 °C and at ambient temperature (28 ± 2 °C) in the storage area of the processing plant.

Three pulp samples (500 g each) were collected into HDPE pouches at the time of pulping and after the heat treatment for testing. Similarly, samples were collected from three random plastic containers (50 kg capacity) during storage from both cold storage (4 °C) and ambient temperature storage at two‐week intervals up to 20 weeks. The collected pulp samples were frozen (−18 °C) until analyses of the vitamin C, polyphenols, Trolox equivalent antioxidant capacity (TEAC), β‐carotene and flavonoids were conducted.

## ANALYSIS OF THE CONSTITUENTS

### Vitamin C

The vitamin C content was analysed by titration with 2,6‐dichlorophenol‐indophenol (DCP) as described by the Association of Official Analytical Chemists (AOAC),^9^ and the values are expressed as grams per kilogram of fresh weight (FW).

### Total polyphenolics

The content of total polyphenols was analysed using the Folin–Ciocalteu reagent as described by Singleton *et al*.^10^ A calibration curve was prepared using gallic acid. Values are expressed in grams of gallic acid equivalents per kilogram fresh weight (g GAE kg^‐1^ FW).

### β‐Carotene

The β‐carotene content was determined by reversed‐phase high‐performance liquid chromatography (RP‐HPLC). The HPLC instrument (Shimadzu, CTO‐10A vp, Kyoto, Japan) consisted of a Vydac 218TP54 (C_18_, 5 μm, 4.6 ID × 250 mm) reversed‐phase analytical column with a guard column. The β‐carotene was extracted using a slightly modified version of the method described by Bushway^11^ and Bushway and Wilson.^12^ The extraction was carried out under dim red light while flushing with nitrogen to minimize oxidation. Two grams of sample and 4 μL of 0.1% butylated hydroxy toluene (BHT) in ethanol were combined with 1.0 mL of internal standard, β‐apo‐8‐carotenal [0.08 mg mL^−1^ in tetrahydrofuran (THF)]. Thereafter, 4 g of anhydrous sodium sulphate, 0.5 g of magnesium carbonate and 30 mL of THF were added. The suspension was mixed using a vortex mixer (VELP Scientifica ZX3 advanced vortex mixer) at 36 g for 1 min, and the mixture was allowed to precipitate to generate a clear supernatant. This supernatant was filtered through filter paper (Whatman No. 1) into a 250 mL round‐bottom flask. Then, the remaining precipitate was extracted with 20 mL of THF as described previously. This procedure was repeated three times until the filtrate and residue were colourless. The filtrate was concentrated until near dryness by a vacuum rotary evaporator (40 °C, ±260 mbar) and flushed with nitrogen. Subsequently, the concentrate was dissolved in 10 mL of a methanol/THF mixture (3:1) containing 0.01% BHT. A 1 mL aliquot was filtered through a 0.45 μm polytetrafluoroethylene (PTFE) HPLC syringe filter (Alltech, Deerfield, IL, USA) into a vial prior to HPLC injection.

The eluent, composed of 92.5% methanol, 7.5% THF and 0.1% triethylamine, was degassed by an Alltech degassing system. A series of samples and standard solutions (20 μL each) were injected into the column and eluted using an isocratic method over 25 min with a flow rate of 1.0 mL min^−1^. β‐Carotene was identified by comparison with an internal standard (β‐apo‐8‐carotenal) and quantified based on its spectrum and peak area.

### Antioxidant activity

The free radical scavenging capacities of different antioxidants in the samples were measured using a DPPH (1,1‐diphenyl‐2‐picrylhydrazyl) assay described by Sánchez‐Moreno *et al*.^13^ and later modified for measuring lipophilic compounds by Jiménez‐Escrig *et al*.^14^ A series of dilutions of the samples of processed fruit products were prepared in methanol.

DPPH (236 mg) was dissolved in 100 mL of methanol; 10 ml aliquots of this solution were prepared and stored at 0 °C. A working solution (6 × 10^−5^ mol L^–1^) was prepared by diluting the stock solution by a factor of 100 in methanol. A standard curve for DPPH was established. The absorption was measured at 515 nm. DPPH solution (6 × 10^−5^ mol L^–1^, 3.9 mL) was carefully transferred into the cuvette, and then 0.1 mL of sample was added. The mixture was kept in the dark at a temperature of 23 ± 1 °C for 30 min, and the absorption was measured at 515 nm. Trolox was used as the standard reference antioxidant.

The amount of DPPH not reacted was determined using the DPPH calibration curve. An efficiency coefficient (EC_50_), the amount of sample necessary to reduce the initial DPPH concentration by 50%, was determined and compared with the value obtained for Trolox. The antioxidant capacity is expressed as the TEAC mmol Trolox kg^‐1^ FW.

### Flavonoid analysis

HPLC analysis was performed to detect the flavonoid compounds. Approximately 5 g of pulp was macerated by adding liquid nitrogen and using a mortar and a pestle. Methanol (5 mL, 100%) was added, and the suspension was left at ambient temperature for 30 min. Mixing was performed in 5 min intervals with a vortex mixer. The suspension was centrifuged at 1308×*g* for 10 min (Himac CT4D Hitachi, Berkshire, UK), and the supernatant was separated. Milli‐Q water brought to pH 2.5 by adding trifluoroacetic acid (TFA) and degassed (Alltech solvent degasser) was used as the elution buffer. Acetonitrile was used as the other eluent. The supernatant of the sample (1 mL) was mixed with 1 mL of Milli‐Q water/TFA (pH 2.5) and filtered through a 0.45 μm PTFE filter (Alltech) into an HPLC vial prior to being taken up into the injection syringe (manual injection) for HPLC analysis.

Standard phenolic compounds, tannic acid, chlorogenic acid, caffeic acid, epicatechin, catechin, and sinapic acid, were used for identification and quantification. The standard stock solutions were prepared by dissolving the compounds in methanol to a concentration of 1 mg mL^−1^, and the solutions were stored at −20 °C.

A Polaris C18 Varian column (SS 150*4.6 mm with a guard column) was used for the measurements, and the detection was performed by UV (wavelength, λ = 220–380 nm). The injection volume was 20 μL, and the flow rate was 1.0 mL min^−1^. The elution buffer, Milli‐Q water acidified to a pH of 2.5 with TFA, and acetonitrile were used. Both elution buffer solutions were sonicated (20 min) before use. The gradient was 0–42% acetonitrile in 20 min; it was held at the final concentration for 5 min; the column was re‐equilibrated for 5 min. The column temperature and the run time were 35 °C and 31 min, respectively.

Quantification was performed by calibration curves, and identification was achieved by comparison of the spectra and retention times of the peaks in the standard solutions with the samples. The quantity of the flavonoid compounds is expressed in mg kg^‐1^ FW.

### Kinetic modelling

Kinetic modelling of the degradation of the bioactive compounds was performed by fitting non‐linear models of the first‐order degradation model to the experimental data.

$$C_{t}=C_{o}e^{-k_{d}\;t}$$

where *C*_*t*_ is the concentration at time *t*, *C*_0_ is the initial concentration and *k*_d_ is the degradation rate constant.

The ratio of the degradation rate constants for mango and pineapple during ambient temperature (28 ± 2 °C) and cold storage (4 °C) were calculated for comparison.

### Statistical analysis

Statistical analysis was conducted using Minitab 16 (version 16) software with one‐way analysis of variance (ANOVA) with Tukey's method to compare the means. Values were considered significant at the level of *P* < 0.05. The confidence interval was 95%. The regression analysis was performed using SPSS 15.0 for Windows. The multivariate statistical method, principal component analysis (PCA) was used to determine how closely health promoting compounds are related to TEAC.

## RESULTS AND DISCUSSION

### Effects of processing on the health‐promoting compounds in mango and pineapple pulp

#### *Fresh fruits*

The values of vitamin C, total polyphenols, TEAC and β‐carotene in ripe mango and pineapple, after pulping, after heat treatment and ready to store pulp (final product) are given in Table 1. The dry matter contents in the ripe mango and pineapple pulps were 14.65% and 12.34%, respectively. The vitamin C content found in mango, 0.24 ± 0.01 g kg^−1^ FW, was lower than the previously reported values of 0.61 ± 0.02 g kg^−1^ FW^3^ and 0.39 ± 0.01 g kg^−1^ FW.^15^ The vitamin C contents vary substantially with changes in variety, cultivar, agronomic practices, postharvest activities, and climatic and soil conditions.

**Table 1:** The content of vitamin C, Trolox equivalent antioxidant capacity (TEAC), polyphenols and β‐carotene, tannic acid, chlorogenic acid, epicatechin and catechin of mango and pineapple at different steps of pulp preparation

	Ripe fruit	Pulp extraction	Heat treatment	Final product
*Mango*				
Vitamin C (g kg^−1^ FW)	0.24 ± .01^a^	0.22 ± 0.01^a^	0.13 ± 0.00^b^	0.12 ± 0.01^b^
Polyphenols (g GAE kg^−1^ FW)	0.34 ± 0.02^a^	0.37 ± 0.02^a^	0.18 ± 0.01^b^	0.16 ± 0.01^b^
TEAC (mmol Trolox kg^−1^ FW)	7.36 ± 0.49^a^	7.51 ± 0.32^a^	4.83 ± 0.51^b^	3.81 ± 0.52^b^
β‐Carotene (×10^−3^ g kg^−1^ FW)	2.42 ± 0.19^a^	2.18 ± 0.18^a^	1.69 ± 0.14^b^	1.45 ± 0.13^b^
*Pineapple*				
Vitamin C (g kg^−1^ FW)	0.39 ± 0.03^a^	0.37 ± 0.02^a^	0.26 ± 0.02^b^	0.24 ± 0.02^b^
Polyphenols (g GAE kg^−1^ FW)	0.38 ± 0.00^a^	0.39 ± 0.00^a^	0.23 ± 0.01^b^	0.22 ± 0.01^b^
TEAC (mmol Trolox kg^−1^ FW)	8.22 ± 0.54^a^	6.95 ± 0.48^b^	5.05 ± 0.39^c^	4.54 ± 0.41^c^
β‐Carotene (×10^−3^ g kg^−1^ FW)	2.38 ± 0.11^a^	2.20 ± 0.12^a^	1.42 ± 0.21^b^	1.36 ± 0.19^b^
*Mango*				
Tannic acid (×10^−3^ g kg^−1^ FW)	68.22 ± 1.92^a^	78.77 ± 2.04^b^	52.36 ± 1.57^c^	48.24 ± 1.39^c^
Chlorogenic acid (×10^−3^ g kg^−1^ FW)	18.85 ± 1.83^a^	28.20 ± 1.83^b^	21.11 ± 1.98^a^	18.84 ± 1.27^a^
Epicatechin (×10^−3^ g kg^−1^ FW)	38.81 ± 1.27^a^	46.65 ± 1.19^b^	32.26 ± 2.00^c^	30.10 ± 1.38^c^
Catechin (×10^−3^ g kg^−1^ FW)	64.07 ± 1.39^a^	78.76 ± 2.16^b^	45.53 ± 1.42^c^	42.22 ± 1.37^c^
*Pineapple*				
Tannic acid (×10^−3^ g kg^−1^ FW)	30.89 ± 1.86^a^	43.35 ± 1.44^b^	32.22 ± 2.09^a^	29.05 ± 1.56^a^
Chlorogenic acid (×10^−3^ g kg^−1^ FW)	46.03 ± 2.06^a^	52.22 ± 1.01^b^	40.03 ± 0.71^c^	38.83 ± 1.48^c^
Epicatechin (×10^−3^ g kg^−1^ FW)	22.41 ± 3.41^ab^	27.62 ± 1.26^a^	18.44 ± 1.72^b^	17.82 ± 2.18^b^
Catechin (×10^−3^ g kg^−1^ FW)	64.51 ± 1.41^a^	68.09 ± 1.51^a^	54.57 ± 3.03^b^	50.43 ± 1.39^b^

Means [± standard deviation (SD)] sharing similar superscripts in a row are statistically non‐significant (*P* < 0.05).

Data expressed as mean value ± SD (*n* = 3). FW, fresh weight.

Final product: thermally treated extracted pulp at ambient (28 ± 2 °C) temperature just after processing and before the storage.

The content of total polyphenols in mango, 0.34 ± 0.03 g GAE kg^−1^ FW, was lower than that reported by Luximon‐Ramma *et al*. 3 (0.56 ± 0.02 g GAE kg^−1^ FW). The TEAC value found in this study, 7.36 ± 0.49 mmol Trolox kg^−1^ FW, was higher than the reported value of 5 mmol Trolox kg^−1^ FW^3^. The measured β‐carotene content (2.42 ± 0.2 mg kg^−1^ FW) in mango was within the range 1.35–18.72 × 10^−3^ g kg^−1^ FW^16^ and close to the reported value 3.08 × 10^−3^ g kg^−1^ FW.^17^

The measured vitamin C content in pineapple (0.39 ± 0.03 g kg^−1^) was higher than the previously reported values of 0.28 g kg^−3^. In fresh pineapple juice, the vitamin C content has been reported to be between 0.092 and 0.94 g L^−1^.^18^, ^19^ The vitamin C content measured in pineapple juice in this experiment was lower than the reported value of 0.84 ± 0.10 g kg^−1^.^18^

The measured content of total polyphenolic compounds (0.38 ± 0.03 g kg^−1^) in pineapple was similar to the values (0.40 ± 0.01 g kg^−1^) reported by Brat *et al*.^20^ Furthermore, higher levels of polyphenolic compounds were also reported by other authors^21^ (0.94 ± 0.02 g GAE kg^−1^ and 0.48 ± 0.01 g GAE kg^−1^).^3^

The measured antioxidant capacity in pineapple (8.2 ± 0.5 mmol Trolox kg^−1^ FW) was similar to the values reported by other authors (9.9 mmol kg^−1^ FW^22^ and 7.2–8.4 mmol kg^−1^ FW^23^). The measured β‐carotene in pineapple, 2.38 ± 0.11 × 10^−3^ g kg^−1^ FW, was similar to the reported value of 2.04 ± 0.19 × 10^−3^ g kg^−1^ FW.^4^

The compositions of flavonoid compounds (tannic acid, chlorogenic acid, epicatechin and catechin) in mango and pineapple pulp at different steps of pulp preparation are given in Table 1. The comparative contents of gallic acid, dietary fibre and total polyphenols in mango and pineapple are reported.^24^ The tannic acid and epicatechin contents in ripe mango flesh were 1.2 and 0.7 times higher than the measured contents of the same compounds in ripe pineapple flesh. The chlorogenic acid content in pineapple was 1.4 times higher than that in mango, while similar contents of catechin were measured in the two fruits (Table 1).

#### *Effect of pulping*

The vitamin C and β‐carotene contents in the fruits measurably decreased during pulping, but the values were not significantly different (*P* > 0.05). In mango, the total polyphenols, 0.37 ± 0.03 g GAE kg^−1^, at the pulping stage were higher than that in the ripe fruit (0.34 ± 0.02 g GAE kg^−1^). The total polyphenols in mango and pineapple increased by 0.03 and 0.02 g GAE kg^−1^, respectively, during the pulp extraction process. Disruption of the cellular matrix in the fruit during pulping facilitated the release of polyphenol compounds. However, the values were not significantly difference (*P* > 0.05) in the vitamin C, polyphenols and β‐carotene contents in the ripe fruits and extracted pulp of mango and pineapple. There was a noticeable decrease in the TEAC value between ripe and extracted pineapple pulp. The difference was significant (*P* < 0.05) for pineapple pulp (Table 1), while that of mango the TEAC value was not significantly changed (*P* > 0.05).

Loss of β‐carotene content at the pulp extraction step alone was 9.9% for mango and 7.5% for pineapple and the difference was not significantly different (*P* > 0.05). These losses could be due to the maceration of the fruit flesh during pulping, which damage the cellular matrix, allowing thermal and oxidative degradation of carotenoids. There was a small increase in the content of flavonoid compounds during the pulp extraction process, which could be due to the maceration of the fruit matrix and the release of flavonoid compounds from the cellular matrix, enhancing the extraction efficiency.

All the flavonoids increased in mango and pineapple pulp except epicatechin and catechin in pineapple (Table 1). There was a significant difference (*P* < 0.05) in between the contents of flavonoids in the ripe fruits and the extracted pulp in both mango and pineapple, except epicatechin and catechin contents in pineapple pulp.

#### *Thermal treatment*

The values of vitamin C, total polyphenols, TEAC and β‐carotene in the extracted pulps of mango and pineapple were significantly decreased (*P* < 0.05) following the heat treatment step in both mango and pineapple pulps. The reduction in the vitamin C content would be due to the oxidation of ascorbic acid into dehydroascorbic or diketogulonic acid during heat treatment.^25^ Pasteurization of pineapple juice at 99 °C for 17 min was reported to cause a 94% loss^26^ and treatment at 90 °C for 3 min caused a 62% loss of vitamin C.^19^

The results revealed significantly (*P* < 0.05) lower values of the bioactive compounds and TEAC in mango and pineapple pulp following the thermal treatment at 85 ± 1 °C for 20 to 25 min in the steam‐jacketed kettle at 100 °C (Table 1). The target entity of the thermal treatment is the oxidative enzymes such as polyphenol oxidase and common spoilage fungi in mango and pineapple namely *Phomopsisi* sp., *Fusarium* sp. and *Penicillium* sp. Further, the bacterial growth in fruit pulp is uncommon due to acidic nature, however the pulp can be contaminated due to cross contamination during pulp extraction by the pathogenic bacteria such as *Escherichia coli* and salmonella sp.^27^ The overall reduction in the vitamin C, total polyphenols, TEAC and β‐carotene values in mango pulp were 42%, 51%, 36% and 23%, respectively. Similarly, the reductions in the same parameters for pineapple pulp were smaller than those in mango except for β‐carotene (vitamin C 29%, total polyphenols 42%, TEAC 27% and β‐carotene 35%). However, a significant difference (*P* < 0.05) in reduction of β‐carotene content in mango (23%) and pineapple (35%) pulps was measured at the thermal treatment step of fruit pulp.

Decreases in the total β‐carotene contents ranging from 7.7% to 15.4% were caused by pasteurization of the mango puree using a holding time of 16 min at temperatures between 85 and 93 °C.^28^ A 27*%* reduction in all‐*trans*‐β‐carotene in pasteurized mango nectar was reported by Vásquez‐Caicedo *et al*.^29^ Reductions in the major carotenoid pigments, β‐carotene (13%), violaxanthin (33%) and luteoxanthin, were reported in homogenized mango puree. These pigments were degraded due to drastic disruption of the ultrastructure of tissues and additional heat treatment at 80 °C (10 min) used in the production of this mango puree.^30^ Therefore, the reductions in the β‐carotene in the mango and pineapple pulps in this experiment were due to the high temperature treatment at 100 °C for 20 to 25 min in the steam‐jacked kettle. A similar treatment is applied in most fruit industries. The application of a more effective method of thermal treatment, such as compatible heat exchangers (scraped surface, tubular, or plate heat exchangers) and vacuum evaporators, might reduce the impact on health‐promoting compounds.

The reductions in the contents of flavonoid compounds in mango and pineapple pulp during heat treatment of the pulps are shown in Table 1. Tannic acid, chlorogenic acid, epicatechin and catechin contents were decreased significantly (*P* < 0.05) during the heat treatment in both the mango and pineapple pulps. In the mango pulp, the decreases in the tannic acid, chlorogenic acid, epicatechin and catechin contents were 34%, 25%, 31% and 42%, respectively, and the decreases in pineapple pulp were 26%, 23% 33% and 20%, respectively. For comparison, the loss of flavonoids in pineapple pulp during heat treatment is lower than that in the mango pulp. All the values of bioactive compounds in the final product (pulp ready to store) are significantly lower than those of ripe fruit, except for chlorogenic acid in mango, tannic acid and epicatechin in pineapple (Table 1). Therefore, the process parameters of the thermal treatment process must be carefully monitored to ensure the quality of the fruit pulp and maintain the maximum levels of flavonoids in the final products.

#### *Bulk storage*

The measured contents of vitamin C, total polyphenols, TEAC and β‐carotene at 20 weeks of storage period are given in Table 2. However, the percentage of losses of each compound at the ambient storage is substantially higher than at the cold storage in mango and pineapple pulps. Percentage of loss of vitamin C, total polyphenols and β‐carotene were 2–2.6, 1.7–1.9 and 1.5–1.7 times higher at the ambient storage than at the cold storage condition in mango and pineapple pulp. Percentage of loss of vitamin C, total polyphenols and β‐carotene in mango (32–54%) were similar in pineapple (37–45%) pulp at cold storage temperature.

**Table 2:** The content of vitamin C, Trolox equivalent antioxidant capacity (TEAC), polyphenols and β‐carotene of mango and pineapple pulp in bulk storage at cold (4 °C) and ambient (28 ± 2 °C) temperature

Weeks	0	2	4	6	8	10	12	14	16	18	20
*Mango pulp in cold storage (4 °C)*											
Vitamin C (g kg^−1^ FW)	0.12 ± 0.00^a^	0.12 ± 0.00^ab^	0.12 ± 0.00^bc^	0.11 ± 0.00^c^	0.11 ± 0.00^d^	0.10 ± 0.00^e^	0.09 ± 0.00^f^	0.09 ± 0.00^fg^	0.09 ± 0.00^fg^	0.08 ± 0.00^g^	0.08 ± 0.00^g^
Polyphenols (gGAE kg^−1^ FW)	0.22 ± 0.00^a^	0.22 ± 0.00^a^	0.20 ± .00^b^	0.20 ± 0.00^b^	0.18 ± 0.00^c^	0.17 ± 0.00^c^	0.17 ± 0.00^c^	0.15 ± 0.00^d^	0.14 ± 0.00^de^	0.14 ± 0.00^e^	0.12 ± 0.00^f^
TEAC (mmol Trolox kg^−1^ FW)	3.21 ± 0.08^a^	3.48 ± 0.04^ab^	3.57 ± 0.04^bc^	3.77 ± 0.16^c^	3.88 ± 0.04^c^	4.15 ± 0.07^cd^	4.24 ± 0.02^de^	4.33 ± 0.04^ef^	4.43 ± 0.04^fg^	4.68 ± 0.11^gh^	4.83 ± 0.04^h^
β carotene (×10^−3^ g kg^−1^ FW)	1.45 ± 0.01^a^	1.22 ± 0.02^b^	1.20 ± 0.07^b^	1.08 ± 0.04^b^	0.88 ± 0.04^c^	0.80 ± 0.00^cd^	0.74 ± 0.02^cde^	0.74 ± 0.02^cde^	0.77 ± 0.01^cde^	0.65 ± 0.06^e^	0.66 ± 0.03^de^
*Mango pulp in ambient storage (28 °C)*											
Vitamin C (g kg^−1^ FW)	0.12 ± 0.00^a^	0.10 ± 0.00^b^	0.10 ± 0.00^b^	0.09 ± 0.00^c^	0.07 ± 0.00^d^	0.04 ± 0.00^e^	0.04 ± 0.01^f^	0.02 ± 0.00^g^	0.02 ± 0.00^h^	13.0 ± 01.4^h^	13.3 ± 00.4^h^
Polyphenols (gGAE kg^−1^ FW)	0.22 ± 0.01^a^	0.19 ± 0.00^b^	0.16 ± 0.00^c^	0.13 ± 0.00^d^	0.11 ± 0.00^e^	0.00 ± 0.00^e^	.0.08 ± 0.00^f^	0.07 ± 0.00^fg^	0.07 ± 0.00^gh^	62.3 ± 00.4^gh^	56.0 ± 02.8^h^
TEAC (mmol Trolox kg^−1^ FW)	3.24 ± 0.05^f^	3.73 ± 0.18^ef^	3.83 ± 0.04^ef^	4.00 ± 0.14^e^	4.38 ± 0.11^e^	5.58 ± 0.11^d^	5.88 ± 0.04^cd^	6.38 ± 0.11^c^	7.90 ± 0.48^b^	8.63 ± 0.18^a^	8.85 ± 0.07^a^
β carotene (×10^−3^ g kg^−1^ FW)	1.41 ± 0.08^a^	1.24 ± 0.02^b^	0.90 ± 0.07^c^	0.83 ± 0.04^cd^	0.68 ± 0.04^de^	0.54 ± 0.02^ef^	0.48 ± 0.04^fg^	0.39 ± 0.01^fgh^	0.35 ± 0.02^gh^	0.33 ± 0.01^gh^	0.27 ± 0.01^h^
*Pineapple pulp in cold storage (4 °C)*											
Vitamin C (g kg^−1^ FW)	0.21 ± 0.00^a^	0.22 ± 0.00^ab^	0.22 ± 0.00^b^	0.18 ± 0.00^c^	0.18 ± 0.00^c^	0.17 ± 0.00^d^	0.16 ± 0.00^d^	0.15 ± 0.00^e^	0.14 ± 0.00^e^	0.14 ± 0.01^e^	0.14 ± 0.00^e^
Polyphenols (gGAE kg^−1^ FW)	0.16 ± 0.00^a^	0.15 ± 0.00^b^	0.13 ± 0.00^c^	0.12 ± 0.01^cd^	0.12 ± 0.00^cd^	0.12 ± 0.01^d^	0.11 ± 0.00^e^	0.10 ± 0.00^ef^	0.10 ± 0.01^ef^	0.11 ± 0.01^f^	0.10 ± 0.00^f^
TEAC (mmol Trolox kg^−1^ FW)	2.55 ± 0.14^f^	2.74 ± 0.08^f^	3.30 ± 0.14^e^	3.75 ± 0.14^d^	3.93 ± 0.04^cd^	4.13 ± 0.04^bc^	4.64 ± 0.02^a^	4.48 ± 0.11^ab^	4.63 ± 0.04^a^	4.78 ± 0.04^a^	4.77 ± 0.09^a^
β carotene (×10^−3^ g kg^−1^ FW)	1.25 ± 0.01^a^	1.30 ± 0.23^a^	1.07 ± 0.18^ab^	0.88 ± 0.04^bc^	0.81 ± 0.01^bc^	0.79 ± 0.04^bc^	0.73 ± 0.01^bc^	0.66 ± 0.03^c^	0.64 ± 0.02^c^	0.62 ± 0.00^c^	0.68 ± 0.01^c^
*Pineapple pulp in ambient storage (28 °C)*											
Vitamin C (g kg^−1^ FW)	0.23 ± 0.01^a^	0.21 ± 0.01^b^	0.19 ± 0.01^c^	0.18 ± 0.00^c^	0.14 ± 0.00^d^	0.13 ± 0.00^e^	0.08 ± 0.00^f^	0.07 ± 0.00^g^	0.05 ± 0.00^h^	0.05 ± 0.00^h^	0.04 ± 0.00^h^
Polyphenols (gGAE kg^−1^ FW)	0.16 ± 0.00^a^	0.14 ± 0.01^b^	0.12 ± 0.00^c^	0.11 ± 0.00^d^	0.09 ± 0.00^e^	0.08 ± 0.00^f^	0.07 ± 0.00^fg^	0.07 ± 0.00^g^	0.06 ± 0.00^h^	0.05 ± 0.00^i^	0.04 ± 0.00^i^
TEAC (mmol Trolox kg^−1^ FW)	2.63 ± 0.04^h^	2.88 ± 0.04^gh^	3.33 ± 0.18^fg^	3.82 ± 0.03^f^	4.35 ± 0.21^e^	4.85 ± 0.07^e^	5.50 ± 0.14^d^	5.95 ± 0.21^cd^	6.30 ± 0.14^bc^	6.52 ± 0.09^ab^	6.83 ± 0.04^a^
β carotene (×10^−3^ g kg^−1^ FW)	1.23 ± 0.04^a^	0.98 ± 0.11^b^	0.90 ± 0.07^b^	0.62 ± 0.03^c^	0.53 ± 0.01^cd^	0.47 ± 0.01^cde^	0.43 ± 0.01^def^	0.37 ± 0.01^defg^	0.32 ± 0.03^efg^	0.28 ± 0.00^fg^	0.26 ± 0.01^g^

Means [± standard deviation (SD)] sharing similar superscripts in a row are statistically non‐significant (*P* < 0.05).

Data expressed as mean value ± SD (*n* = 3). FW, fresh weight.

The levels of tannic acid, chlorogenic acid, epicatechin and catechin measured in mango and pineapple pulp stored at cold and ambient temperatures are shown in Table 3. Measurable quantities of flavonoids remained in the mango and pineapple pulps after 20 weeks at 4 °C. During ambient temperature storage, tannic acid (2.4 ± 0.0 mg kg^−1^ FW, at 16th week), chlorogenic acid (1.4 ± 0.1 mg kg^−1^ FW, at 10th week), epicatechin (1.1 ± 0.2 mg kg^−1^ FW, at 12th week) and catechin (1.7 ± 0.2 mg kg^−1^ FW at 18th week) were detected in mango pulp. Furthermore, in mango pulp tannic acid, chlorogenic acid and epicatechin were not detected after 16th, 10th and 12th weeks at ambient temperature of storage whereas pineapple pulp, tannic acid and epicatechin were not detected after the 14th and 12th weeks, respectively, at ambient temperature of storage.

**Table 3:** The content of tannic, chlorogenic, epicatechin and catechin of mango and pineapple pulp in bulk storage at cold (4 °C) and ambient (28 ± 2 °C) temperature

Weeks	0	2	4	6	8	10	12	14	16	18	20
*Mango pulp in cold storage (4 °C)*											
Tannic (×10^−3^ g kg^−1^ FW)	48.43 ± 0.18^a^	45.95 ± 0.49^ab^	44.29 ± 0.05^bc^	41.78 ± 0.67^c^	37.55 ± 1.56^d^	32.25 ± 0.35^e^	25.43 ± 1.31^f^	18.58 ± 0.11^g^	14.58 ± 0.11^h^	9.8 ± 0.57^i^	6.6 ± 0.28^j^
Chlorogenic (×10^−3^ g kg^−1^ FW)	18.18 ± 0.54^a^	14.4 ± 0.28^b^	12.58 ± 0.32^c^	10.68 ± 0.17^d^	7.9 ± 0.41^e^	7.57 ± 0.09^e^	6.3 ± 0.14^f^	4.7 ± 0.41^g^	3.15 ± 0.07^h^	2.65 ± 0.21^hi^	1.76 ± 0.15^i^
Epicatechin (×10^−3^ g kg^−1^ FW)	29.4 ± 1.56^a^	23.93 ± 0.60^b^	21.08 ± 0.81^c^	17.05 ± 0.78^d^	14.9 ± 0.42^de^	12.45 ± 0.07^e^	8.58 ± 0.11^f^	6.35 ± 0.14^fg^	4.31 ± 0.06^gh^	2.85 ± 0.07^hi^	1.33 ± 0.18^i^
Catechin (×10^−3^ g kg^−1^ FW)	41.93 ± 0.18^a^	40.74 ± 0.72^a^	35.53 ± 1.43^b^	31.87 ± 0.52^c^	26.17 ± 0.52^d^	21.91 ± 0.58^e^	17.60 ± 0.85^f^	12.09 ± 0.36^g^	9.09 ± 0.69^h^	5.40 ± 0.28^i^	4.56 ± 0.34^i^
*Mango pulp in ambient storage (28 °C)*											
Tannic (×10^−3^ g kg^−1^ FW)	48.28 ± 0.39^a^	42.15 ± 0.71^b^	31.55 ± 1.27^c^	25.59 ± 1.79^d^	20.84 ± 0.73^e^	17.50 ± 1.41^e^	11.43 ± 1.31^f^	7.52 ± 1.39^f^	2.38 ± 0.04^g^	ND	ND
Chlorogenic ((×10^−3^ g kg^−1^ FW)	18.58 ± 0.03^a^	15.91 ± 0.49^b^	13.05 ± 0.78^c^	8.53 ± 0.11^d^	4.40 ± 0.41^e^	1.40 ± 0.14^f^	ND	ND	ND	ND	ND
Epicatechin (×10^−3^ g kg^−1^ FW)	31.00 ± 0.71^a^	26.93 ± 0.60^b^	20.90 ± 0.85^c^	15.88 ± 0.67^d^	8.43 ± 0.32^e^	4.03 ± 0.32^f^	1.05 ± 0.21^g^	ND	ND	ND	ND
Catechin (×10^−3^ g kg^−1^ FW)	41.79 ± 0.37^a^	35.88 ± 0.81^b^	28.78 ± 0.31^c^	24.86 ± 0.71^d^	17.96 ± 0.56^e^	11.84 ± 0.73^f^	7.95 ± 0.49^g^	4.22 ± 0.02^h^	3.05 ± 0.21^hi^	1.65 ± 0.21^i^	ND
*Pineapple pulp in cold storage (4 °C)*											
Tannic (×10^−3^ g kg^−1^ FW)	30.85 ± 0.49^a^	29.00 ± 0.71^ab^	21.15 ± 0.49^b^	23.58 ± 1.10^c^	18.90 ± 0.85^d^	18.50 ± 0.41^d^	12.70 ± 0.41^e^	8.40 ± 0.00^f^	4.55 ± 0.07^g^	3.60 ± 0.28^g^	2.95 ± 0.21^g^
Chlorogenic (×10^−3^ g kg^−1^ FW)	38.05 ± 0.64^a^	34.25 ± 0.35^b^	31.00 ± 0.28^c^	28.55 ± 0.07^d^	25.20 ± 0.57^e^	21.40 ± 0.41^f^	19.00 ± 0.71^g^	15.05 ± 0.64^h^	12.54 ± 0.09^i^	8.50 ± 0.41^j^	5.90 ± 0.41^k^
Epicatechin (×10^−3^ g kg^−1^ FW)	17.23 ± 0.60^a^	15.60 ± 0.28^b^	13.08 ± 0.81^c^	11.40 ± 0.07^d^	9.64 ± 0.23^e^	7.73 ± 0.11^f^	6.47 ± 0.02^f^	4.27 ± 0.08^g^	2.45 ± 0.07^h^	1.22 ± 0.02^hi^	0.95 ± 0.21^i^
Catechin (×10^−3^ g kg^−1^ FW)	49.88 ± 0.53^a^	48.05 ± 0.64^ab^	46.41 ± 0.13^bc^	44.33 ± 0.75^cd^	42.48 ± 0.25^d^	36.59 ± 1.37^e^	31.95 ± 0.42^f^	25.48 ± 1.30^g^	18.58 ± 0.03^h^	12.48 ± 0.18^i^	8.33 ± 0.11^j^
*Pineapple pulp in ambient storage (28 °C)*											
Tannic (×10^−3^ g kg^−1^ FW)	31.00 ± 0.71^a^	26.00 ± 0.57^b^	21.48 ± 0.04^c^	18.05 ± 0.78^d^	12.53 ± 0.11^e^	8.39 ± 0.38^f^	4.36 ± 0.21^g^	1.43 ± 0.32^h^	ND	ND	ND
Chlorogenic (×10^−3^ g kg^−1^ FW)	38.35 ± 0.21^a^	35.42 ± 0.26^b^	31.33 ± 0.18^c^	25.43 ± 0.31^d^	18.38 ± 0.04^e^	11.91 ± 0.58^f^	8.55 ± 0.07^g^	6.60 ± 0.28^h^	4.53 ± 0.39^i^	3.33 ± 0.18^ij^	2.15 ± 0.07^j^
Epicatechin (×10^−3^ g kg^−1^ FW)	17.08 ± 0.81^a^	12.55 ± 0.41^b^	8.53 ± 0.10^c^	5.42 ± 0.30^d^	3.49 ± 0.41^e^	1.43 ± 0.32^f^	0.85 ± 0.07^f^	ND	ND	ND	ND
Catechin (×10^−3^ g kg^−1^ FW)	49.88 ± 0.53^a^	45.93 ± 1.03^b^	41.43 ± 0.32^c^	38.25 ± 0.35^d^	31.98 ± 0.53^e^	25.50 ± 1.41^f^	20.43 ± 1.31^g^	14.85 ± 0.49^h^	11.08 ± 0.39^i^	8.50 ± 0.14^ij^	6.50 ± 0.42^j^

Means [± standard deviation (SD)] sharing similar superscripts in a column are statistically non‐significant (*P* < 0.05).

Data expressed as mean value ± SD (*n* = 3). FW, fresh weight.

However, there were losses of 6.5% to 11% in the tannic acid, chlorogenic acid, epicatechin and catechin contents in mango pulp, whereas in pineapple pulp, the losses were only 3–9.5%. These losses could be due to the exposure of the pulp to ambient air during handling and the effect of light.

The quantitative loss of vitamins could be predicted based on an accurate understanding of the kinetics and temperature dependence of the particular form(s) of the vitamin(s) in the food matrices. A vitamin consists of different chemical forms that react differently based on the composition of the food and the specific processing conditions.^31^ The results revealed that the current practice of ambient temperature storage is not suitable from a nutritional perspective because the health‐promoting compounds are not retained in the final products. Measurably higher quantities of the components were retained during storage at 4 °C.

Although pulp sensory properties can be retained during ambient temperature storage, making it a cost‐effective method, the retention of health‐promoting compounds is poor. Therefore, the quantities of health‐promoting compounds in final products derived from pulp/juice stored at ambient temperature were very low or negligible compared to those derived from materials stored cold. Therefore, the potential health benefits of products processed using bulk stored pulp at 28 ± 2 °C would be far lower compared to products produced from fresh fruit pulp or juice.

#### *Degradation rate constants*

**Figure 1:**
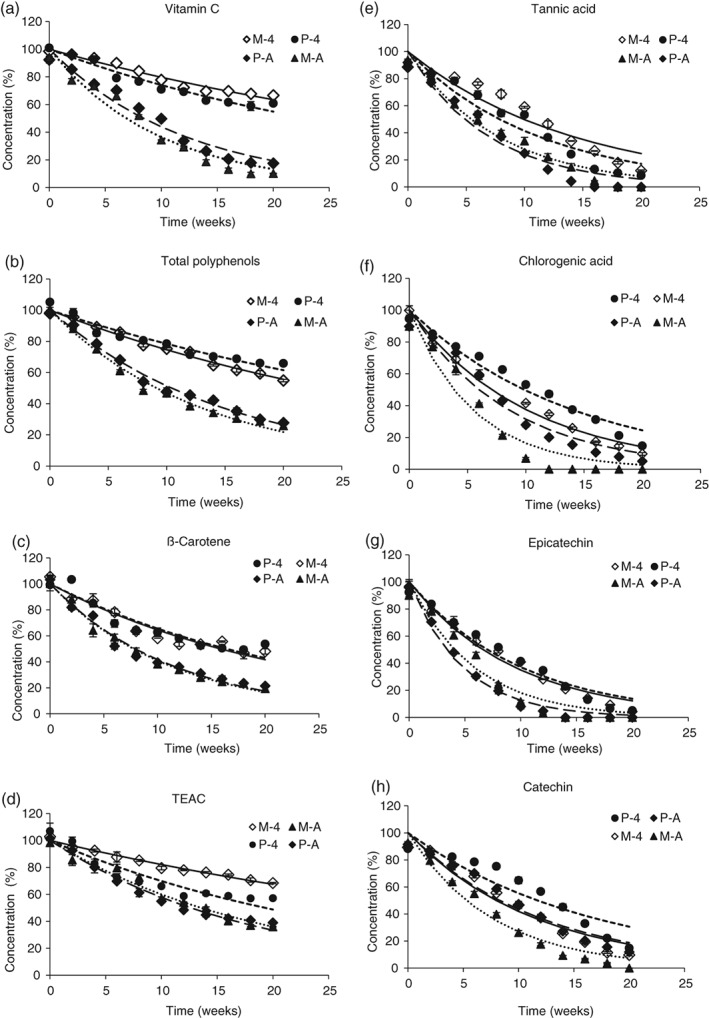
Concentration (%) *versus* time (weeks) in bulk stored mango (M 4, 4 °C; M‐A ambient storage) and pineapple (P‐4, 4 °C; P‐A ambient storage) pulp. The solid and dotted lines represent the respective model of the measured data.

The degradation rate constants (*k*_d_ values) of vitamin C, total polyphenols, TEAC and β‐carotene in stored mango and pineapple pulp stored at 4 °C and ambient temperature (28 ± 2 °C) are given in Table 4. The degradation rate constants of vitamin C, total polyphenols, TEAC and β‐carotene in mango pulp stored cold were significantly lower than those in mango pulp stored at ambient temperature. Concentration (%) *versus* time (in weeks) in bulk stored mango and pineapple pulps at 4 °C and ambient storage (28 ± 2°C) is give in Fig. 1. The health‐promoting compounds degraded faster in the pulp stored at ambient temperature then pulp stored at 4 °C. Of the samples tested, mango pulp stored at 4 °C showed the slowest rate of TEAC degradation (Fig. 1(d)). The higher temperatures accelerate the degradation and loss of the compounds through chemical reactions in the pulp. Similarly, the degradation rate constants of pineapple pulp stored cold were lower for the degradation rate constant than those of the pineapple pulp stored at ambient temperature (Table 4).

**Table 4:** Degradation rate constant of health promoting compounds during the pulp storage

		Degradation rate *k*_d_ (week^‐1^)			
Type of pulp	Storage condition	Vitamin C	Polyphenols	TEAC	β‐Carotene
Mango	4 °C	2.27 × 10^‐2^ (2.05 × 10^‐2^ –2.48 × 10^‐2^)	2.93 × 10^‐2^ (2.76 × 10^2^–3.10 × 10^‐2^)	1.97 × 10^‐2^ (1.80 × 10^‐2^–2.13 × 10^‐2^)	4.38 × 10^‐2^ (3.79 × 10^‐2^–4.98 × 10^‐2^)
Mango	Ambient 28 ± 2 °C	1.01 × 10^‐1^ (8.80 × 10^‐2^ –1.14 × 10^‐1^)	7.61 × 10^‐2^ (7.13 × 10^‐2^ –8.08 × 10^‐2^)	5.12 × 10^‐2^ (4.65 × 10^‐2^–5.59 × 10^‐2^)	8.96 × 10^‐2^ (8.34 × 10^‐2^–9.58 × 10^‐2^)
Pineapple	4 °C	3.00 × 10^‐2^ (2.69 × 10^‐2^ –3.31 × 10^‐2^)	2.43 × 10^‐2^ (2.12 × 10^‐2^ –2.75 × 10^‐2^)	3.59 × 10^‐2^ (2.96 × 10^‐2^–4.22 × 10^‐2^)	4.23 × 10^‐2^ (3.34 × 10^‐2^–5.12 × 10^‐2)^
Pineapple	Ambient 28 ± 2 °C	8.24 × 10^‐2^ (7.28 × 10^‐2^–9.19 × 10^‐2^)	6.65 × 10^‐2^ (6.28 × 10^‐2^–7.03 × 10^‐2^)	5.49 × 10^‐2^ (4.86 × 10^‐2^–6.12 × 10^‐2^)	8.76 × 10^‐2^ (7.92 × 10^‐2^–9.60 × 10^‐2^)

		Tannic	Chlorogenic	Catechin	Epicatechin
Mango	4 °C	6.99 × 10^‐2^ (5.66 × 10^‐2^–8.32 × 10^‐2^)	9.81 × 10^‐2^ (9.18 × 10^‐2^–1.04 × 10^‐1^)	8.69 × 10^‐2^ (7.48 × 10^‐2^–9.90 × 10^‐2^)	1.05 × 10^‐1^ (9.47 × 10^‐2^–1.15 × 10^‐1^)
Mango	Ambient 28 ± 2 °C	1.28 × 10^‐1^ (1.12 × 10^‐1^–1.44 × 10^‐1^)	1.81 × 10^‐1^ (1.47 × 10^‐1^–2.16 × 10^‐1^)	1.31 × 10^‐1^ 1.15 × 10^‐1^–1.48 × 10^‐1^	1.71 × 10^‐1^ (1.41 × 10^‐1^–2.01 × 10^‐1^)
Pineapple	4 °C	8.83 × 10^‐2^ (7.28 × 10^‐2^–1.04 × 10^‐1^)	7.04 × 10^‐2^ (6.27 × 10^‐2^–7.82 × 10^‐2^)	5.92 × 10^‐2^ (4.63 × 10^‐2^–7.22 × 10^‐2^)	9.92 × 10^‐2^ (8.62 × 10^‐2^–1.12 × 10^‐1^)
Pineapple	Ambient 28 ± 2 °C	1.43 × 10^‐1^ (1.21×10^‐1^–1.65 × 10^‐1^)	1.16 × 10^‐1^ (1.00 × 10^‐1^–1.31 × 10^‐1^)	8.35 × 10^‐2^ (7.29 × 10^‐2^–9.41 × 10^‐2^)	2.07×10^‐1^ (1.89 × 10^‐1^–2.26 × 10^‐1^)

Confidence interval is given within brackets (95%).

According to the ratios of the degradation rate constants, the vitamin C, polyphenols, TEAC and β‐carotene in mango pulp deteriorate 4.5, 2.6, 2.6 and 2.0 times faster than when stored at ambient temperature, and for pineapple pulp, the deterioration rates were 2.7, 2.7, 1.5 and 2.1 times greater, respectively. The results revealed that the degradation rate of β‐carotene at ambient temperature was almost similar in both fruit pulps.

The degradation rate constants (*k*_d_ values) of tannic acid, chlorogenic acid, epicatechin and catechin in mango and pineapple pulp during storage are given in Table 4. Similar to the other compounds, the degradation rates of flavonoids in both mango and pineapple pulps were high during storage at ambient temperature. The degradation of the flavonoid compounds in the mango pulp was 1.5–1.8 times faster under ambient storage. Furthermore, the degradation rates of tannic acid, chlorogenic acid, epicatechin and catechin during ambient temperature storage of pineapple pulp were 1.6, 1.6, 2.1 and 1.4 times greater than those in cold storage.

#### *Correlation coefficients*

**Figure 2:**
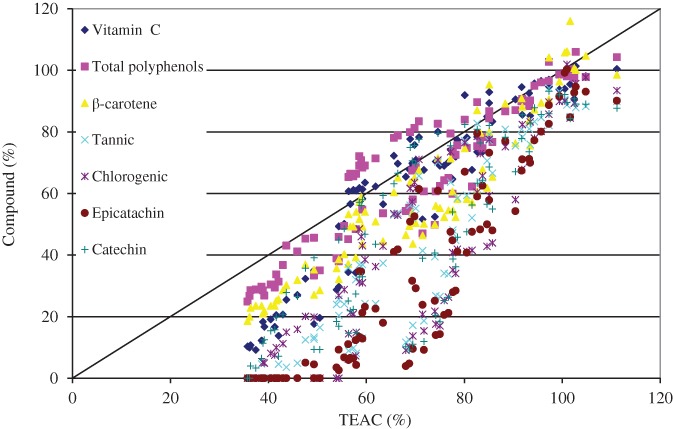
Correlation between the Trolox equivalent antioxidant capacity (TEAC) (%) and the health‐promoting compounds (%).

For all the samples measured in this study, the concentrations of vitamin C, total polyphenols, β‐carotene, tannic acid, chlorogenic acid, epicatechin, and catechin were plotted against their TEAC percentage (Fig. 2). The TEAC values were correlated with the vitamin C (*R* ^2^ = 0.93), polyphenols (*R* ^2^ = 0.92), β‐carotene (*R* ^2^ = 0.94), tannic acid (*R* ^2^ = 0.93) and epicatechin (*R* ^2^ = 0.91) contents. However, chlorogenic acid, catechin and other compounds possessed relatively lower coefficients. Figure 2 shows that even at zero concentrations of these species, the TEAC percentage remains high. Therefore, vitamin C, polyphenols and β‐carotene show behaviours similar to TEAC over the whole range of TEAC percentage.

Contribution of the bioactive compounds of tropical fruits on the antioxidant activity was reported by Luximon‐Ramma *et al*.^3^. TEAC values of 1 to 47 mmol Trolox kg^‐1^ FW, total phenolic contents of 0.118 to 5.638 g kg^‐1^ FW, proanthocyanidin contents of 0.007 to 2.561 g kg FW and vitamin C contents of 0.008 to 1.426 g kg in 17 commonly consumed exotic Mauritian fruits were reported.^3^. The regression analysis between the antioxidant activity (TEAC and FRAP) *versus* the total phenolics (*R* ^2^ = 0.98 and *R* ^2^ = 0.95), total flavonoids (*R* ^2^ = 0.77 and *R* ^2^ = 0.69), and total proanthocyanidin (*R* ^2^ = 0.96 and *R* ^2^ = 0.92) contents were reported. There were strong correlations between the antioxidant activity [TEAC and fluorescence recovery after photobleaching (FRAP)] and the total phenolics and proanthocyanidins contents. The contributions of flavonoids to the antioxidant potentials of these fruits were substantially lower. The contribution of one or more phytochemicals to the antioxidant capacity is evident. The correlation coefficient between TEAC and vitamin C in this experiment was *R* ^2^ = 0.93, which could be due to the effect of a high content of vitamin C or the potential antioxidant activities of other phytochemicals present in the fruit matrix.

The formation of reaction products that may also have antioxidant activities can interfere in analyses of antioxidant activity; these interferences may introduce errors in the evaluation of correlations between individual parameters.^32^ In this study, a good correlation coefficient (*R* ^2^ = 0.92) was found between the total polyphenols content and the antioxidant activity (TEAC). Linear correlations between the total polyphenols and the antioxidant activities [oxygen radical absorbance capacity (ORAC) values] have been reported previously for fruits.^33^ The correlation coefficient (*R* ^2^ = 0.97), between the total phenolics content and the cupric reducing antioxidant activity (CUPRAC) was reported by Kamiloglu and Capanoglu.^34^

#### *Principal component analysis (PCA)*

**Figure 3:**
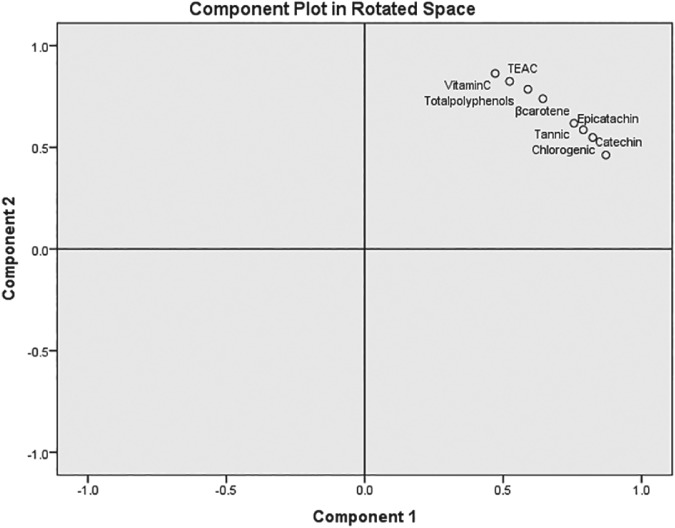
Component plot in rotated space. Components 1 and 2 represent 92.7% and 3.7% of variance, respectively.

Multivariate data analysis using PCA with varimax rotation was conducted on TEAC values and vitamin C, total polyphenols, β‐carotene, tannic acid, chlorogenic acid, epicatechin and catechin using SPSS. Two components that explained 96.4% of the variance were identified. The PCA plot of the two components is given in Fig. 3. According to Fig. 3, all variables are clustered together and thus all phytochemicals are closely associated with TEAC. Furthermore, according to Fig. 3, the vitamin C, total polyphenols, and β‐carotene variables are closer to TEAC, and it can be attributed to the fact that these compounds contribute more to the TEAC than tannic acid, epicatechin, chlorogenic acid and catechin.

When the same PCA was performed for each temperature of each fruit types (mango and pineapple), the general pattern of relationship between TEAC and other variables were found to be more or less the same as for the overall case (Fig. 3); i.e. all variables clustered in one quadrant. However minor deviations of pattern of relationship were observed in the individual plots. For pineapple pulp**,** at 4 °C, the closest variable to TEAC was β carotene followed by total polyphenols. In ambient temperature, for pineapple pulp, the closest variable was total polyphenols.

When plots were obtained for each fruit type regardless of temperature, still the same pattern was observed. However, the minor deviation observed was owing to the fact that vitamin C was the closest compound to TEAC followed by total polyphenol for mango, and epicatechin for pineapple. When plots were obtained regardless of fruits type, a similar pattern was observed. Although vitamin C was the closest compound to TEAC under cold store of pulp, under ambient temperature, vitamin C was located to TEAC after tannic acid and epicatechin.

## CONCLUSIONS

The total polyphenol content in mango and pineapple pulps increased by 0.025 and 0.021 g GAE kg^‐1^ FW during the pulping step. There were significant reductions in the vitamin C, total polyphenols, TEAC and β‐carotene values in both mango and pineapple pulp during the heat treatment at 100 °C for 20 to 25 min; the reductions were relatively smaller for pineapple. Based on multivariate PCA, vitamin C, total polyphenols, and β‐carotene values are closer to the TEAC values. Thus, these compounds contributed more to the TEAC than tannic acid, epicatechin, chlorogenic acid and catechin; however, the latter compounds also show strong contributions.

The ratio of the degradation rate constants (*k*_d_ values) of vitamin C, polyphenols, TEAC and β‐carotene during storage at ambient temperature compared to those at 4 °C ranged between 2 and 4.5 for mango pulp and between 1.5 and 2.7 for pineapple pulp. The bulk storage of pulps at cold temperatures (4 °C) provided better retention of health‐promoting compounds than ambient temperature storage (28 ± 2 °C) for up to 20 weeks. Therefore, a storage temperature of 4 °C is recommended for the bulk storage and preservation of mango and pineapple pulps. The authors recommend the shelf‐life of thermally treated bulk stored mango and pineapple pulps should be determined based on the health promoting compounds namely vitamin C, total polyphenols, and β‐carotene.

## CONFLICT OF INTEREST

The authors have no conflicts of interest to disclose.
